# LAPAROSCOPIC SPLENIC FLEXURE MOBILIZATION: TECHNICAL ASPECTS, INDICATION CRITERIA AND OUTCOMES

**DOI:** 10.1590/0102-672020210001e1575

**Published:** 2021-06-11

**Authors:** Fabio Guilherme CAMPOS, Leonardo Alfonso BUSTAMANTE-LOPEZ, Carlos Augusto MARTINEZ

**Affiliations:** 1 University of São Paulo, Faculty of Medicine, Department of Gastroenterology and Coloproctology, São Paulo, SP, Brazil; 2 University São Francisco, Faculty of Medicine, Department of Surgery, Bragança Paulista, São Paulo, SP, Brazil

**Keywords:** Colorectal surgery, Laparoscopy, General surgery, Cirurgia colorretal, Laparoscopia, Cirurgia geral

## INTRODUCTION

The laparoscopic treatment of colorectal diseases has brought many technical and tactical modifications as an attempt to improve results over open surgery. In the context, the growing experience with laparoscopic techniques^1^ ^-^ ^5^ allowed the adoption of a complete splenic flexure mobilization (SFM) as an essential step during colorectal resections^6^. This maneuver aims to ensure a tension-free and well-perfused length of colon to be attached at the anastomosis, allowing an adequate resection margin in segmental left resections for diverticulitis or cancer.

There exist some controversies regarding laparoscopic SFM, such as selective indication, the best moment to perform, the need for additional ports and technical aspects. Furthermore, there is a common fear that an additional procedure could affect postoperative morbidity.

A literature search shows that laparoscopic SFM lacks standardization. Consequently, we decided to describe in detail important technical aspects of how it can be performed and to discuss indications and outcomes.

## METHOD

This study was submitted and approved by the institutional ethics committee of São Paulo University, São Paulo, SP, Brazil under n^o^. 9076078.

### Surgical technique step by step

#### Preparing the operative field

**FIGURE 1:**
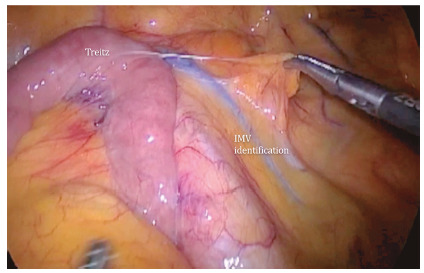
Identification and traction of the inferior mesenteric vein (IMV) near the duodenojejunal ligament (Treitz); this is the first step in mobilizing splenic flexion.

The patient is settled in a modified Lloyd-Davies position with Trendelenburg. We prefer to introduce five ports: 10 mm umbilical for the camera and four others in each abdominal quadrant (12 mm trocar in the right iliac fossa). The falciform ligament is transected, so the great omentum and transverse colon are lifted over the liver and secured with a grasper introduced in the right upper quadrant (Figure 1). After this, the surgeon moves from a position between legs to the right side of the patient.

#### Inferior mesenteric vein dissection

**FIGURE 2:**
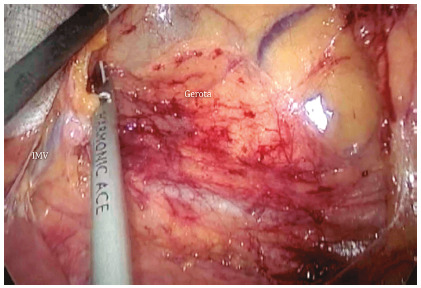
Medial-lateral dissection that exposes the left renal fascia (Gerota´s) under the pancreas and progresses laterally towards Toldt’s fascia

The next step is inferior mesenteric vein identification near the duodenojejunal flexure (Treitz, Figure 2). The grasper inserted in the left upper abdominal quadrant holds the vein so the surgeon may dissect the avascular plane beneath the vein and over the left renal fascia (Gerota’s). A medial-to-lateral dissection defines an area limited superiorly by the pancreas border, laterally by the fusion fascia of Toldt (Toldt’s fascia) and inferiorly by the inferior mesenteric artery emergence (Figure 2). Finally, the inferior mesenteric vein is transected using energy devices or clips.

#### Mesocolon dissection

**FIGURE 3:**
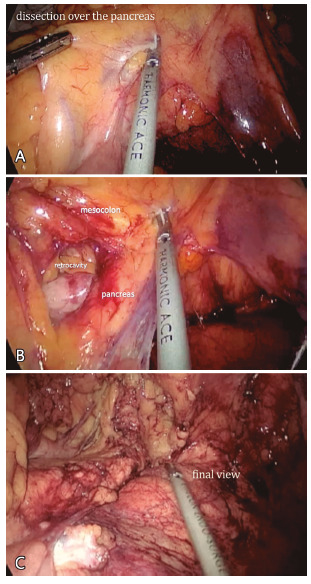
A) Retroperitoneal space is accessed through an area located around 3-5 cm above and to the left from the duodenojejunal flexure (Treitz); B) subsequently, the dissection progresses carefully over the anterior surface of the pancreas towards the tail, releasing the transverse mesocolon from its posterior attachments; C) finally, the transverse mesocolon is completely separated from the retroperitoneum.

Transverse colon traction facilitates the identification of an area located around 3-5cm superiorly and to the left from duodenojejunal flexure, where a gentle perforation facilitates the access to the retroperitoneum (Figure 3A). At this moment, one may see the posterior gastric wall. Subsequently, dissection progresses carefully over the anterior surface of the pancreas towards its tail, thus relieving the transverse mesocolon from its posterior attachments (Figures 3B and 3C).

#### Transverse colon mobilization

**FIGURE 4:**
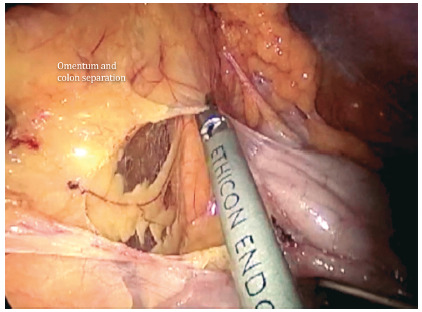
Proper exposure allows the surgeon to separate the large colon omentum (intercoloepiploic detachment), progressing from transverse colon towards the splenic flexure, to obtain mobilization

Appropriate exposure allows the surgeon to separate the great omentum from the colon (intercoloepiploic detachment), coming from the transverse colon towards the splenic flexure (Figure 4). Finally, the lateral attachments of the descending colon at the Toldt’s fascia are dissected to achieve complete take down of the splenic flexure.

## DISCUSSION

Technically, a confortable colon descensus towards the pelvis depends on the level of vascular control and on the detachment of embryological fusions to the retroperitoneum or pancreas. Routine or selective indications of SFM have been a great source of controversy among surgeons. Most of them agree that it is indispensable during low anterior resection or coloanal anastomosis, especially when the construction of a colonic pouch is considered necessary. Another common indication is diverticulitis^9^, due to the associated inflammation and the need to resect the sigmoid or the descending colon^3^. On the contrary, if it is perceived that confortable tension-free anastomosis is possible due to a redundant colon, it may be avoided^7^ ^,^ ^10^.

In a different view, the choice of a routine SFM is based on the small increment in the procedure length (around 10% of the total time) and on the low risk of complications such as splenic injury. As the associated morbidity is extremely rare, the only disadvantage of SFM is its learning curve. Thus, most laparoscopic surgeons prefer to start surgery performing SFM primarily, a decision that may allow an eventual ischemia to become apparent at the extreme end of the proximal colon before constructing the anastomosis.

Moreover, an early SFM have also the advantage of avoiding a disappointing situation at the end of the procedure, in cases in which some tension is perceived when descending the colon for anastomosis^8^. Additionally, SFM provides a chance to obtain a more vascularized colonic end at the anastomosis, an effect that may compensate some ischemia due to the inferior mesenteric artery high ligation. 

Many surgeons prefer to do it in a routine fashion (and we agree with this position in our department), based on the idea that initial technical difficulties may be easily surpassed with practice.
